# Altered Functional Connectivity of the Basal Nucleus of Meynert in Mild Cognitive Impairment: A Resting-State fMRI Study

**DOI:** 10.3389/fnagi.2017.00127

**Published:** 2017-05-04

**Authors:** Hui Li, Xiuqin Jia, Zhigang Qi, Xiang Fan, Tian Ma, Hong Ni, Chiang-shan R. Li, Kuncheng Li

**Affiliations:** ^1^Department of Radiology, Xuanwu Hospital, Capital Medical UniversityBeijing, China; ^2^Beijing Key Lab of MRI and Brain InformaticsBeijing, China; ^3^Department of Psychiatry, Yale University School of MedicineNew Haven, CT, USA; ^4^Department of Neuroscience, Yale University School of MedicineNew Haven, CT, USA; ^5^Beijing Huilongguan HospitalBeijing, China

**Keywords:** basal nucleus of Meynert, basal forebrain, cholinergic bases, mild cognitive impairment, functional connectivity

## Abstract

**Background**: Cholinergic dysfunction plays an important role in mild cognitive impairment (MCI). The basal nucleus of Meynert (BNM) provides the main source of cortical cholinergic innervation. Previous studies have characterized structural changes of the cholinergic basal forebrain in individuals at risk of developing Alzheimer’s disease (AD). However, whether and how functional connectivity of the BNM (BNM-FC) is altered in MCI remains unknown.

**Objective**: The aim of this study was to identify alterations in BNM-FC in individuals with MCI as compared to healthy controls (HCs), and to examine the relationship between these alterations with neuropsychological measures in individuals with MCI.

**Method**: One-hundred-and-one MCI patients and 103 HCs underwent resting-state functional magnetic resonance imaging (rs-fMRI). Imaging data were processed with SPM8 and CONN software. BNM-FC was examined via correlation in low frequency fMRI signal fluctuations between the BNM and all other brain voxels. Group differences were examined with a covariance analysis with age, gender, education level, mean framewise displacement (FD) and global correlation (GCOR) as nuisance covariates. Pearson’s correlation was conducted to evaluate the relationship between the BNM-FC and clinical assessments.

**Result**: Compared with HCs, individuals with MCI showed significantly decreased BNM-FC in the left insula extending into claustrum (insula/claustrum). Furthermore, greater decrease in BNM-FC with insula/claustrum was associated with more severe impairment in immediate recall during Auditory Verbal Learning Test (AVLT) in MCI patients.

**Conclusion**: MCI is associated with changes in BNM-FC to the insula/claustrum in relation to cognitive impairments. These new findings may advance research of the cholinergic bases of cognitive dysfunction during healthy aging and in individuals at risk of developing AD.

## Introduction

Mild cognitive impairment (MCI), as a syndrome of cognitive decline without demonstrable alteration in daily activities (Gauthier et al., 2006), frequently precedes the development of Alzheimer’s disease (AD; Petersen, 2011). Epidemiological studies reveal that 3% to 19% of the adults older than 65 years of age suffer from MCI (Gauthier et al., 2006). Most of the patients with MCI experience poor memory as the primary symptom (Kawas, 2003). Memory formation and maintenance is supported by a wide swath of cerebral structures and endogenous acetylcholine plays a critical role in the modulation of information acquisition, encoding, consolidation and retrieval (Blokland et al., 1992; Boccia et al., 2003, 2004, 2009; Power et al., 2003; Winters and Bussey, 2005). As the most important component of the basal forebrain cholinergic system (BFCS; Liu et al., 2015), the basal nucleus of Meynert (BNM) provides main sources of cholinergic innervation to the cerebral cortex (Gratwicke et al., 2013). In MCI, β-amyloid (Aβ) deposition, neurofibrillary tangles and trophic support reduction impair cholinergic functions of the BNM (Mesulam et al., 1986; Ruberg et al., 1990; Vogels et al., 1990). It is plausible that dysfunctional BNM-FC may contribute to cognitive decline in patients with MCI (Whitehouse et al., 1981; Mesulam et al., 1983; Nordberg, 1993; Cullen et al., 1997; Grothe et al., 2010; Ferreira-Vieira et al., 2016).

In humans the BNM is located in the BFCS with an extent of approximately 13–14 mm anterio-posteriorly and 16–18 mm medio-laterally (Gratwicke et al., 2013). The small volume and irregular border has impeded research of the BNM. Extant imaging studies have focused on volume changes in the BNM in association with MCI (Hanyu et al., 2002; Zaborszky et al., 2008; Grothe et al., 2012) with reduced volume extending into the whole BFCS in patient with AD (George et al., 2011; Grothe et al., 2013, 2014), as compared with healthy aging (Kilimann et al., 2014). For instance, volume reduction of the BNM and temporal lobe structures was associated with impairment in delayed recall in MCI patients (Grothe et al., 2010). These findings are consistent with neuronal loss in the BNM during the prodromal stage of AD and greater neuronal loss in the BNM than other cortical and subcortical structures in advanced AD (Arendt et al., 2015).

Investigators have suggested that functional alterations likely precede structural atrophy and examination of cerebral functional connectivity may be critical to understanding the etiologies of many neuropsychiatric disease (Greicius et al., 2004; Fox and Raichle, 2007; Qi et al., 2010; Liang et al., 2011). Low-frequency fluctuations of blood oxygenation level–dependent (BOLD) signal that occur during rest reflect connectivity between functionally related brain regions (Biswal et al., 1995; Fair et al., 2007; Fox and Raichle, 2007). Studies of this “spontaneous” activity describe the intrinsic functional architecture of the sensory, motor, cognitive systems (Fox and Raichle, 2007; Manza et al., 2015; Kann et al., 2016; Zhang et al., 2016) and may provide useful information on network functional integrity. For instance, numerous studies have demonstrated altered resting state functional connectivity (rsFC) in patients with MCI and AD (Wang et al., 2006; Sorg et al., 2007; Liang et al., 2012). Compared with healthy controls (HCs), selected areas of the default mode network, including the posterior cingulate, and the executive attention network demonstrated reduced network-related activity in individuals with MCI (Sorg et al., 2007). Patients with mild AD demonstrated disruption in hippocampal connectivity to the medial prefrontal cortex, ventral anterior cingulate cortex, posterior cingulate cortex, right superior and middle temporal gyrus (Wang et al., 2006). The network dysconnectivity appears to aggravate as the illness progresses.

On the other hand, whether or how BNM-FC is altered in MCI or AD remains unknown. On the basis of a previous study that delineated the BNM of post-mortem human brains in a standard stereotaxic space (Zaborszky et al., 2008), we recently characterized whole-brain rsFC of the BNM in a large cohort of healthy adults (Li et al., 2014). Furthermore, we showed that BNM connectivity to the visual and somatomotor cortices decreases while connectivity to subcortical structures including the midbrain, thalamus and pallidum increases with age (Li et al., 2014). These findings of age-related changes of cerebral functional connectivity of the BNM may facilitate research of the neural bases of cognitive decline in health and illness. Here, we pursued this issue by investigating whole-brain rsFC of the BNM in individuals with MCI. We hypothesized that, in comparison with age-matched control participants, BNM connectivity with cortical-subcortical regions would be disrupted in MCI patients in association with cognitive dysfunction.

## Materials and Methods

### Subjects and Assessments

One-hundred and one MCI patients were recruited from the Xuanwu Hospital of Capital Medical University in Beijing. MCI patients met the core clinical criteria stipulated by the National Institute on Aging and the Alzheimer’s Association (Albert et al., 2011) that include: (a) complaint of a change in cognition; (b) impairment in cognitive function, especially episodic memory; (c) ability to maintain independence in daily activities; (d) not demented; and (e) Clinical Dementia Rating (CDR) score = 0.5, with a score of at least 0.5 on the memory domain (Petersen et al., 2001).

One-hundred and three age- and gender-matched HCs were recruited from the community for comparison. The criteria for the HCs was as follows: (a) no current or previous diagnosis of any neurological or psychiatric disorders; (b) no neurological deficiencies in physical examinations; (c) absence of abnormal findings on brain MRI; (d) no complaints of cognitive changes; and (e) a CDR score of 0. Additional exclusion criteria for both MCI and HCs participants included contraindications for MRI such as use of cardiac pacemakers and claustrophobia.

All participants underwent history-taking, complete physical examination and neuropsychological evaluation with Mini-Mental State Examination (MMSE), CDR, Montreal Cognitive Assessment (MoCA) and Auditory Verbal Learning Test (AVLT). MMSE has been a widely used cognitive test (Nilsson, 2007) since its publication in 1975 (Folstein et al., 1975), with excellent sensitivity and moderate specificity in the diagnosis of dementia in specialist settings (Mitchell, 2009). MoCA is a brief, stand-alone cognitive screening tool with high sensitivity and specificity for the detection of MCI and excellent test-retest reliability (Nasreddine et al., 2005). CDR is used to stage the severity of AD in longitudinal studies and clinical trials covering six cognitive categories, including memory, orientation, judgment and problem solving, community affairs, home and hobbies and personal care (Morris, 1993). AVLT provides a standardized procedure to evaluate verbal learning and memory of supra-span lists of words (Volkmar, 2013) and has been proved to be useful in the diagnosis of MCI and could predict disease progression (Zhao Q. et al., 2015). Demographic and assessment data of the participants are shown in Table 1.

**Table 1:** **Clinical characteristics of the mild cognitive impairment (MCI) patients and healthy controls (HCs)**.

Characteristics	HCs (*n* =103)	MCI (*n* =101)	*p*-value
Age (years)	62.79 ± 9.26	65.13 ± 9.36	0.074
Gender (male/female)	40/63	52/49	0.069
Education (years)	11.84 ± 4.09	10.34 ± 4.13	<0.05
CDR	0	0.5	<0.001
Framewise displacement	0.119 ± 0.057	0.126 ± 0.062	0.382
MMSE	28.44 ± 1.61	25.40 ± 3.05	<0.001
MoCA	26.29 ± 2.01	20.68 ± 3.30	<0.001
AVLT-immediate recall	9.13 ± 1.97	6.42 ± 1.86	<0.001
AVLT-delayed recall	9.72 ± 2.78	4.83 ± 3.76	<0.001
AVLT-recognition	11.68 ± 2.82	8.15 ± 3.84	<0.001

*Note: Values represent means ± standard deviation; MMSE, Mini-Mental State Examination; CDR, Clinical Dementia Rate; MoCA, Montreal Cognitive Assessment; AVLT, Auditory Verbal Learning Test; P values were derived from the Student *t*-test comparing the 2 groups except for “gender” where the P value was obtained using the *χ*^2^ test*.

The study was conducted under a research protocol approved by the Ethics Committee of the Xuanwu Hospital, in accordance with the Declaration of Helsinki. All participants were given a detailed explanation of the study and signed an informed consent prior to the study.

### MRI Data Acquisition

MRI data were acquired with a 3-Tesla Trio scanner (Siemens, Erlangen, Germany). All participants were asked to hold still, with their eyes closed. Foam padding was employed to limit head motion and headphones were used to reduce scanner noise. Resting-state functional magnetic resonance imaging (rs-fMRI) images were acquired using an echo-planar imaging (EPI) sequence with a repetition time (TR)/echo time (TE)/flip angle (FA) = 2000 ms/40 ms/90, field of view (FOV) = 256 mm, 28 axial slices, slice thickness/gap = 4/1 mm, bandwidth = 2232 Hz/pixel and number of repetitions = 239. The 3D T1-weighted anatomical image was acquired with a magnetization-prepared rapid gradient echo (MPRAGE) method with the following parameters: TR/TE/inversion time (TI)/FA = 1900 ms/2.2 ms/900 ms/9, bandwidth = 199 mm, matrix = 256 × 224, 176 sagittal slices with 1 mm thickness.

### MRI Data Preprocessing and Functional Connectivity Analysis

Rs-fMRI data were preprocessed using the statistical parametric mapping software SPM8 (Wellcome Department of Imaging Neuroscience, London, UK) and seed-to-voxel correlation analysis was carried out by the functional connectivity (CONN) toolbox v17a (Whitfield-Gabrieli and Nieto-Castanon, 2012). The first 10 functional images were discarded to reduce the fluctuation of MRI signal in the initial stage of scanning. The remaining 229 images of each individual subject were first corrected for slice timing to reduce the within-scan acquisition time differences between slices and then realigned to eliminate the influence of head motion during the experiment. All subjects included in the present study exhibited head motion less than 1.5 mm in any of the x, y, or z directions and less than 1.5° of any angular dimension and volume-level mean framewise displacement (FD) less than 0.30 (with a mean FD across all subjects of 0.12 ± 0.06; Power et al., 2012). Next, the realigned images were spatially normalized to the Montreal Neurological Institute (MNI) space and resampled them to 2 × 2 × 2 mm^3^. Subsequently, the functional images were smoothed with a 4-mm full width at half maximum (FWHM) isotropic Gaussian kernel. After preprocessing, images were then band-pass filtered to 0.008 ~0.09 Hz to reduce the influence of noise. Further denoising steps included regression of the six motion parameters and their first-order derivatives, regression of white matter and cerebrospinal fluid (CSF) signals following the implemented CompCor strategy (Behzadi et al., 2007) and a linear detrending. In second-level covariance analysis, the covariates included age, gender, education level, mean FD (Power et al., 2015), as well as global correlation (GCOR; Saad et al., 2013). Localization of brain region was conducted with xjView^1^.

The seed BNM was, as defined by Li et al. (2014), based on a stereotaxic probabilistic maps of the basal forebrain, that contains the magnocellular cholinergic corticopetal projection neurons (Zaborszky et al., 2008). In the latter study, after a T1-weighted MRI scan, 10 human postmortem brains were made into histological serial sections and stained by silver. The positions and the extent of each part of the basal forebrain were microscopically delineated, 3D reconstructed and warped to the reference space of the MNI brain. Magnocellular cell groups in the subcommissural-sublenticular region of the basal forebrain were defined as the BNM (Vogels et al., 1990; de Lacalle et al., 1991). To consider the influence of volumetric difference in BNM between patients with MCI and HCs, we compared gray matter volume of BNM between MCI and HCs groups.

The correlation coefficients between the seed voxels and all other brain voxels were computed to generate correlation maps. For group analyses the correlation coefficients were converted to *z*-value using Fisher’s *r*-to-*z* transformation to improve normality (Lowe et al., 1998).

### Statistical Analysis

Clinical data and neuropsychological measures were analyzed using SPSS 19, with the Student’s *t*-test conducted for continuous variables and the chi-squared test for dichotomous variables. The BNM-FC maps were analyzed based on a voxel-wise comparison across the whole brain. The individual *z* value was entered into a random effect one sample *t-test* to determine brain regions showing significant connectivity to the BNM within each group. Results within-group were thresholded at voxel-level *p* < 0.05 (FWE corrected) and cluster size >100 voxels. Two-sample *t*-test was performed to compare BNM-FC between MCI and HCs with age, gender, education level, mean FD, as well as GCOR as nuisance covariables. For between-group comparisons, we used 3dClustSim program in AFNI (version: December 8, 2016^2^) to conduct the multiple comparison corrections within a group GM mask (obtained by a threshold of 0.2 on the mean GM probability map of all subjects) using a voxel-wise threshold of *p* < 0.0005 (uncorrected) and cluster size >69 voxels, which corresponded to a corrected *p* < 0.05 (using AFNI’s updated autocorrelation function estimation; Cox et al., 2017).

### Correlation Analysis

Region-of-interest (ROI) analysis was performed on the regions showing significant BNM-FC changes in MCI as compared to HCs. For each subject the mean BNM-FC across all voxels of each ROI was extracted and computed. Partial correlation analysis was then conducted to evaluate the relationship between the BNM-FC to these ROIs and raw scores of clinical assessments, controlled for age, gender, education level, mean FD, as well as GCOR. Statistical significance was set at *p* < 0.05, Bonferroni corrected for multiple comparisons.

## Results

### Demography and Neuropsychological Assessment

As shown in Table 1, no significant difference in age, gender and FD was found between the patients with MCI and HCs. Compared to HCs, MCI patients showed significantly lower education level (*p* < 0.05) and cognitive decline in the MMSE (*p* < 0.001), MoCA (*p* < 0.001) and AVLT (*p* < 0.001) score. Meanwhile, MCI patients showed significantly higher CDR score, as compared with HCs (*p* < 0.001).

### Whole Brain BNM-FC in HCs and MCI Patients

**Figure 1:**
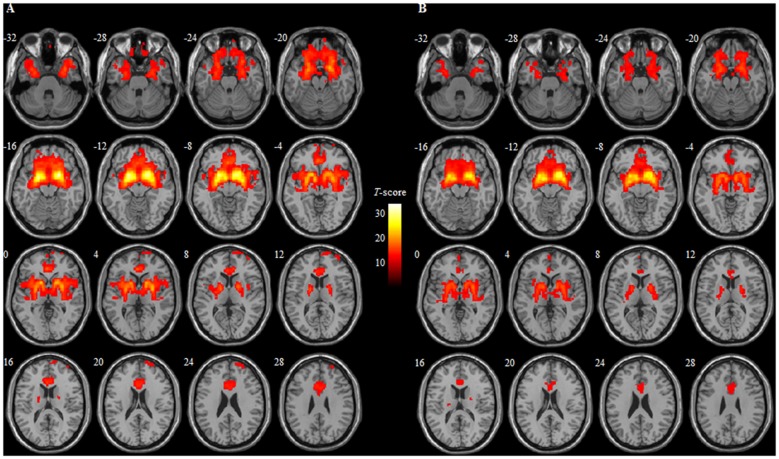
**Whole brain functional connectivity of the basal nucleus of Meynert (BNM) in healthy controls (HCs) (A)** and mild cognitive impairment (MCI) **(B)**. Numbers in the figure indicate the *Z* coordinate in Montreal Neurological Institute (MNI); results within-group were thresholded at voxel-wise* p* < 0.05 (FWE corrected) and cluster size >100 voxels. BNM, basal nucleus of Meynert; colorbar indicates *t*-score.

Within group analysis revealed that the positive connectivity between the BNM and many brain regions both in HCs and individuals with MCI. These regions included the bilateral frontal lobe extending into the orbital/rectal cortex, medial/inferior/middle gyrus, temporal lobe extending into (para) hippocampus/amygdala, basal ganglia including the caudate/putamen/claustrum, as well as thalamus (Table 2 and Figure 1).

**Table 2:** **Regions of basal nucleus of Meynert-functional connectivity (BNM-FC) from one-sample *t* test of each group and regions showing decreased BNM-FC in MCI as compared to HCs**.

Region	Cluster size	Peak coordinate (MNI)			*T*-score
		*x*	*y*	*z*	
**HCs**					
Frontal/Temporal/Limbic/	22,435	18	6	−16	32.93
BG/Thalamus
Rt.Superior frontal gyrus	438	12	68	12	7.28
**MCI**					
Frontal/Temporal/Limbic/	14,285	22	12	−12	27.96
BG/Thalamus
**HCs > MCI**					
Lt.Insula/Claustrum	87	−36	6	2	3.91

*Note: Results within-group were thresholded at voxel-wise* p* < 0.05 (FWE corrected) and cluster size >100 voxels. Results between-group were corrected for multiple comparisons using an uncorrected *p* < 0.0005 and cluster size >69 voxels to yield a corrected *p* < 0.05 (using AFNI’s updated autocorrelation function estimation). BG, basal ganglia; Lt, left; Rt, right*.

### Altered BNM-FC in MCI Patients Compared to HCs

**Figure 2:**
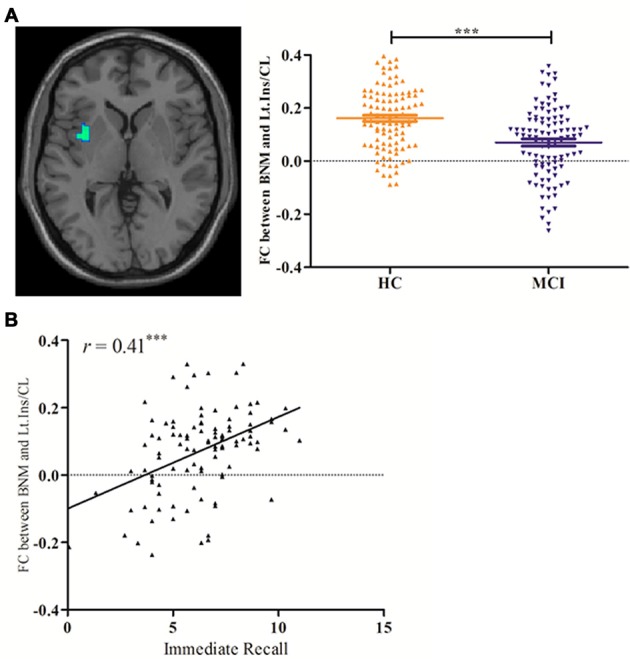
**(A)** Decreased BNM-left insula/claustrum connectivity in MCI patients; and **(B)** linear correlation of BNM-left insula/claustrum connectivity and AVTL-immediate recall performance in MCI patients. BNM, basal nucleus of Meynert; Ins/CL, insula/claustrum; AVLT, Auditory Verbal Learning Test; FC, functional connectivity; ***indicates *p* < 0.0001.

Compared with HCs, significantly decreased BNM-FC was detected in the left insula/claustrum in MCI patients (Figure 2A and Table 2). No brain regions showed increased BNM-FC in MCI patients when compared to HCs. In addition, no significant difference was observed in the gray matter volume of BNM between MCI and HCs (*p* = 0.067).

### Correlation of BNM-FC with Neuropsychological Scores

Pearson’s correlation showed that BNM-FC with the left insula/claustrum was positively associated with the memory performances measured by AVTL-immediate recall (*r* = 0.41, *p* < 0.0001) in MCI patients (Figure 2B). On the other hand, no significant correlation was detected between BNM-FC and neuropsychological measures in HCs.

## Discussion

The present study examined the BNM-FC in MCI patients in comparison to HCs in a relatively large sample of participants. The findings showed decreased BNM-FC at the left insula/claustrum in MCI patients, as compared to HCs. Furthermore, decreased BNM-insula/claustrum functional connectivity was positively associated with impaired performance as measured by the AVLT- immediate recall in MCI patients.

The insula plays a critical role in cognition, emotion, sensory processing and autonomic control (Naqvi et al., 2007; Allen et al., 2008; Kurth et al., 2010). It is also involved in integrating somatosensory, autonomic and cognitive-affective information to guide behavior (Christopher et al., 2014) and switching brain network activities to support various aspects of cognitive functions (Seeley et al., 2007). Studies in monkeys showed that the density of acetylcholinesterase containing fibers (Mesulam et al., 1984) and choline acetyltransferase activities are particularly rich in the insula (Mesulam et al., 1986). Previous studies revealed that the insula was affected in individuals with MCI (Xie et al., 2012) and at risk of developing AD (Foundas et al., 1997) and insula atrophy effectively discriminated AD patients from the healthy populations (Fan et al., 2008; Insel et al., 2015). The Aβ plaques, neurofibrillary tangles and significant volume atrophy have all been reported in the insula in MCI and AD patients (Karas et al., 2004; Braak et al., 2006; He et al., 2015; Ting et al., 2015; Zhao Z. L. et al., 2015). Furthermore, whole-brain correlational analyses revealed that cognitive performance was associated with the volume (Farrow et al., 2007; Lu et al., 2016; Tillema et al., 2016), functional activity and intrinsic connectivity of the insula in MCI patients (Xie et al., 2012; Nellessen et al., 2015).

The striatum, including claustrum, putamen/pallidum and caudate (Chikama et al., 1997) are of central importance to cognitive motor functions (Christopher et al., 2015). The claustrum is an important albeit less studied part of the striatum. Previous studies reported that the claustrum was activated during episodic memory retrieval in HCs (Schwindt and Black, 2009). Recent work demonstrated that the claustrum receives input from multiple brain regions such as the parietal and the medial temporal structures (Park et al., 2012; Torgerson et al., 2015), while projecting to the insula and frontal pole (Burman et al., 2011). Via this anatomical connectivity, the claustrum may play an important role in binding multiple sources of information to facilitate memory-guided behavior. The claustrum showed neurofibrillary, amyloid pathology (Morys et al., 1996) and reduced choline acetyltransferase (Ohara et al., 1996, 1999; Gill et al., 2007) in both MCI and AD. Wang et al. (2016) reported that disrupted amygdala connectivity with the claustrum in AD patients. Thus, in line with these studies, the present work reveals the existence of altered BNM-FC at the claustrum in MCI patients.

Greater decrease in BNM-FC with insula/claustrum was associated with lower verbal episodic memory scores in MCI patients. The claustrum and the insula are functionally and structurally connected (Chikama et al., 1997). The striatum receives insular projections primarily to support motivated behavior, including reward processing and approach and avoidance learning (Chikama et al., 1997). The perisylvian division of the cholinergic fiber bundles originating from BNM traveled within the claustrum and supplied innervations to the insula (Selden et al., 1998). Previous studies revealed that the insula/claustrum is a crucial hub of the brain network integrating information from multiple brain regions through its extensive reciprocal connections to neocortical, limbic and paralimbic structures (Crick and Koch, 2005; Fernández-Miranda et al., 2008; Park et al., 2012; Torgerson et al., 2015). More recently, Seo and Choo (2016) demonstrated positive correlations between memory performance and regional cerebral glucose metabolism in bilateral claustrum and the left insula in MCI. A single photon emission computed tomography study also reported significant correlation between verbal memory test performance and brain perfusion in the left insula and claustrum in AD patients (Nobili et al., 2007). Thus, the positive correlation between verbal episodic memory scores and the BNM-FC with the left claustrum/insula suggests decline in episodic memory in MCI patients in association with the inefficient information integration and decreased functional connectivity at the insula and claustrum.

## Conclusion

In conclusion, MCI patients present significant changes of BNM-FC at the left insula and the claustrum in association with immediate recall during AVLT. These new findings may advance research of the cholinergic bases of cognitive dysfunction during healthy aging and in individuals at risk of developing AD.

## Limitations

Several limitations need to be considered for this study. First, those are a cross-sectional study finding and a longitudinal study is needed to understand how BNM-FC may be related to disease progression. Longitudinal follow-up of the patients would allow us to examine whether these neural phenotypes could predict onset of AD in this MCI cohort. Second, the matching of MCI and HCs groups were marginally significant. Although these factors were included as covariates in data analyses, we could not rule out their potential impact on the current results. Finally, human brains are highly varied among different demographics (e.g., gender, age and race). A recent study has demonstrated that the Chinese brain atlas improved accuracy and reduced anatomical variability during registration (Liang et al., 2015). In the current study, the data of participants were normalized to MNI152. Future studies involving Chinese populations should be considered to normalize to Chinese 2020 (a typical statistical Chinese brain template).

## Author Contributions

HL and XJ carried out data collection and analysis and wrote the manuscript. ZQ helped with data interpretation. XF, TM and HN carried out data collection. CRL contributed to conceptualization of the study and revision of the manuscript. KL contributed to conceptualization and design of the study and revised the manuscript.

## Conflict of Interest Statement

The authors declare that the research was conducted in the absence of any commercial or financial relationships that could be construed as a potential conflict of interest.

## Footnotes

^1^http://www.alivelearn.net/xjview

^2^http://afni.nimh.nih.gov/pub/dist/doc/program_help/3dClustSim.html
